# Effects of continuous biochar application on soil chemical properties and tomato yield and quality in an arched shed

**DOI:** 10.3389/fpls.2025.1666930

**Published:** 2025-10-24

**Authors:** Chitao Sun, Rongxing Niu, Gang Cao, Di Feng

**Affiliations:** ^1^ Graduate Innovation College of Saline-Alkali Land Comprehensive Utilization, Shandong Agricultural University, Taian, China; ^2^ National Center of Technology Innovation for Comprehensive Utilization of Saline-Alkali Lands, Dongying, Shandong, China; ^3^ Institute of Agricultural Resources and Environment, Xinjiang Academy of Agricultural Sciences, Urumchi, China; ^4^ Department of Protected Agriculture Science and Engineering, Weifang University of Science and Technology, Shouguang, China

**Keywords:** biochar, tomato, greenhouse cultivation, fruit quality, soil chemical properties

## Abstract

Biochar is a promising soil amendment, but its long-term consecutive effects on greenhouse tomato systems are insufficiently explored. To investigate the dynamic and accumulative effects of consecutive biochar application on soil chemical properties and tomato (*Solanum lycopersicum* L.) yield and quality, a three-year arched shed field experiment was conducted with five biochar rates: 0 (CK), 0.5 (T1), 1.0 (T2), 2.0 (T3), and 4.0 (T4) kg·m^-^². Soil chemical properties, tomato growth, yield components, and fruit quality were analyzed. Results showed biochar slightly increased soil electrical conductivity (all below salinization threshold); only T4 significantly raised soil pH (by 0.4 units) and organic matter (by 132.8%) *vs*. CK. Annual differences in soil available potassium diminished to non-significance, while available phosphorus was 50.8% (T2) and 63.0% (T3) higher than CK. Tomato plant height and dry matter increased with biochar rate; T1-T4 improved fruits per plant (2.0%-17.0%) and single fruit weight (7.0%-16.0%) over CK, with T2 (13.7%-24.1%) and T3 (19.8%-33.2%) achieving the highest significant yield increases. For quality, T2 had the highest comprehensive index, followed by T1 and T3, with their three-year average scores up by 33.1%, 15.4%, and 15.4% respectively. In conclusion, 1.0-2.0 kg·m^-^² biochar optimally enhanced tomato yield and nutritional/organoleptic quality, with no significant interannual cumulative effects of biochar rate on yield or quality—providing theoretical and technical support for high-quality greenhouse tomato production.

## 1 Introduction

Tomato (*Solanum lycopersicum* L.), the world’s second most consumed vegetable crop (Hasnain et al., 2020), possesses both significant economic value and nutritional functions. Due to its rich nutrient composition and diverse culinary applications, it occupies a core position in the global vegetable industry (Samui et al., 2020). According to statistics from the Food and Agriculture Organization (FAO, 2023), global tomato production reached 186.1 million metric tons in 2022, with a harvested area of 4.7 million hectares. China accounts for approximately 1.119 million hectares of tomato cultivation, of which about 60% is under protected cultivation (FAO, 2023). Tomatoes serve as a primary dietary source of antioxidant compounds such as Vitamin C (VC) and lycopene (Luo et al., 2021), whose intake is closely associated with the prevention of chronic diseases (e.g., cancer, cardiovascular diseases) (Rattanavipanon et al., 2021). In agricultural production, achieving synergistic improvement of high yield and superior quality has always been a core objective in the field of protected tomato cultivation.

Biochar, as one of the key soil amendments for building sustainable agricultural systems (Semida et al., 2019), can be produced via pyrolysis technology from carbon-containing feedstocks such as lignocellulosic biomass, crop straws, livestock manure, and sewage sludge (Bolan et al., 2021). This thermochemical process occurs under low-oxygen or anoxic conditions, primarily generating non-condensable syngas, condensable liquid fractions, and solid product biochar (Osman et al., 2022). The amendment of biochar significantly influences fundamental soil chemical properties such as pH and electrical conductivity (EC). Due to its inherent alkaline nature and the presence of alkaline ash, biochar application typically increases soil pH, which is particularly beneficial for acidic soil remediation (Zhang et al., 2025). Concurrently, the dissolution of salts from biochar and the enhanced release of ions due to the pH increase can elevate soil EC (Mao et al., 2024; Łuczak et al., 2021). However, the extent of these changes is highly dependent on the properties of both the biochar and the native soil. Understanding these dynamics is crucial for assessing the applicability of biochar in specific agricultural contexts, such as protected cultivation. In agriculture, biochar improves soil environment through multiple mechanisms: it not only reduces nutrient leaching, enhances soil fertility (Rosa et al., 2024), and decreases heavy metal bioavailability (Fu et al., 2024), but also improves water-holding capacity by optimizing soil pore structure (Wu et al., 2025), and enhances root nutrient uptake efficiency by promoting mycorrhizal fungal colonization and microbial community diversity (Gu et al., 2021). These properties provide multi-dimensional promotion for crop growth. Additionally, returning pyrolyzed agricultural waste to fields not only achieves by-product recycling but also strengthens the sustainability of agricultural ecosystems. Therefore, biochar application in tomato cultivation can be regarded as a win-win strategy.

In tomato production, biochar application has been found to promote root development, increase root length and fine root proliferation, and enhance nutrient absorption capacity (He et al., 2021), with significant yield improvements observed in some studies (Agbna et al., 2017). However, existing research conclusions are inconsistent: some field trials show no yield increase or even negative effects of biochar on tomatoes (Wu et al., 2022; Obadi et al., 2023). Regarding fruit quality, contradictory results exist in its regulatory effects on total soluble solids (TSS) and vitamin C (VC) content (Abdelghany et al., 2023). Notably, current studies mostly focus on the dosage effects of biochar in a single growing season (Agbna et al., 2017; Guo et al., 2021), but there is a serious lack of understanding of the accumulation characteristics of biochar effects under long-term continuous application.

Given this, the present study conducted a three-year fixed-site experiment on protected tomato cultivation to systematically investigate the dynamic effects and accumulative effects of consecutive biochar application rates (0-4.0 kg·m^-^²) on yield components, fruit quality indices, and soil chemical properties. The objectives were to quantify the response patterns of tomato yield and quality to different application rates and determine the optimal biochar application rate that balances high yield and superior quality. The research findings will provide technical parameters for the precise application of biochar in protected tomato production, facilitating the establishment of a sustainable production model that synergistically improves yield and quality while integrating environmental and economic benefits.

## 2 Materials and methods

### 2.1 Experimental site description

**Figure 1:**
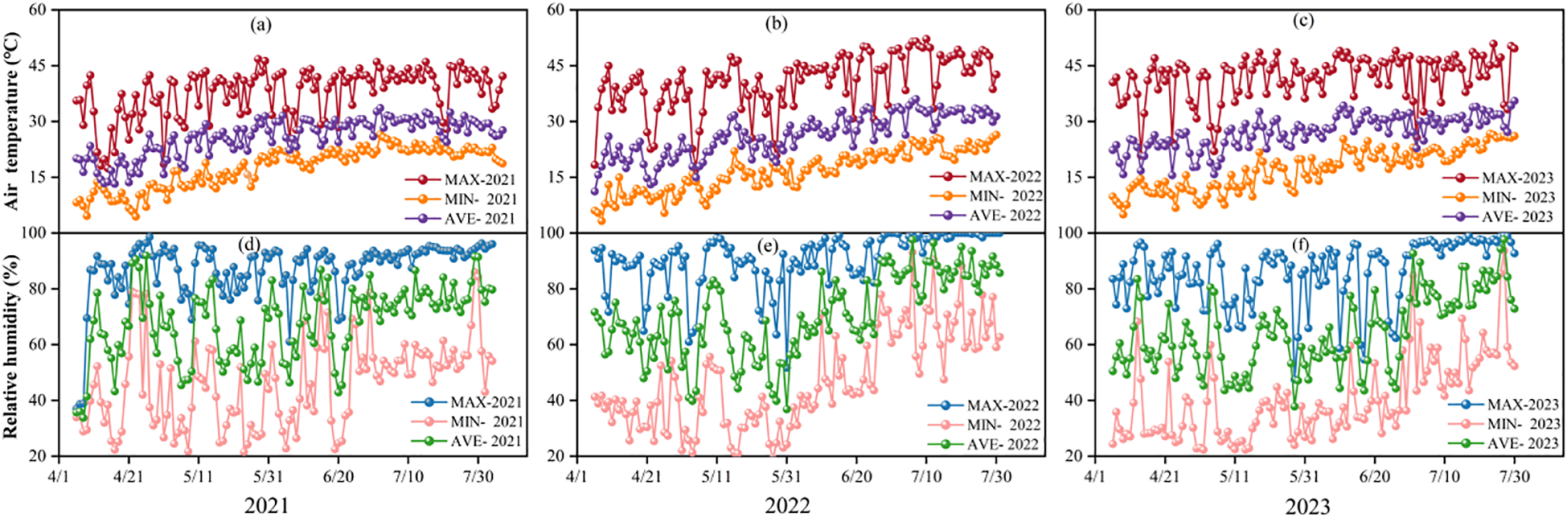
Trends in air temperature **(a-c)** and relative humidity **(d-f)** in protected facilities during 2021-2023.

This study was conducted in a greenhouse tunnel in Shouguang City, Shandong Province (118°44′ E, 36°53′ N) from 2021 to 2023. Tomato cultivar 'Diana' (Wanglin Agriculture, China) was used as the test crop. The biochar applied in the experiment was produced by Pingdingshan Lvzhiyuan Activated Carbon Co., Ltd., using a mixture of crop straws, wood chips, and fruit shells as raw materials. The biochar was pyrolyzed at 600°C and then ground into 50–100 mesh powder. The soil type in the experimental area was loam, and the basic chemical properties and nutrient status of the soil and biochar are detailed in **Table 1**. The fertilizer used was a compound fertilizer (N-P_2_O_5_-K_2_O ≥ 60%, 20-20-20 + Fe+Zn+B ≥ 0.2%) produced by Jinzhengda Ecological Engineering Group Co., Ltd. The variations in air temperature and relative humidity inside the facility during the experimental period are presented in **Figure 1**. During the growing season, the mean air temperature and mean relative humidity were 25.7°C and 67.4% in 2021, 26.0°C and 70.1% in 2022, and 27.1°C and 64.2% in 2023, respectively. Overall, the diurnal temperature range narrowed gradually over the three experimental years. Notably, the variation in day-night temperature difference was smaller in 2021 than in 2022 and 2023.

**Table 1:** Physical and chemical properties of tested soil.

Item	Total N	Available P	Available K	Organic matter	Hydrolyzable N	Salt content	pH
Unit	g·kg^-1^	mg·kg^-1^	mg·kg^-1^	g·kg^-1^	mg·kg^-1^	%	
Soil	0.84	7.75	127.50	12.90	52.00	0.04	7.41
Biochar	1.16	665.00	508.00	80.60	20.40	0.36	7.77

### 2.2 Experimental design

The trial adopted a tomato (April-August) and Chinese cabbage (August-December) rotation system. A single-factor randomized block design was implemented with five biochar application rates (0, 0.5, 1.0, 2.0, and 4.0 kg·m^-^²), labeled as CK (control), T1, T2, T3, and T4, respectively. The plot dimensions were 3.0 m × 1.2 m, with two rows of tomato plants per plot, spaced at 60 cm row spacing and 30 cm plant spacing, totaling 20 plants per plot. Biochar was uniformly broadcast and incorporated into the 0–10 cm soil layer annually before tomato seedling transplantation, while no biochar was applied during the cabbage cultivation phase. A drip irrigation system with 16 mm diameter tubing, emitters spaced at 30 cm intervals, and a flow rate of 2 L·h^-^¹ was installed immediately after transplanting tomato seedlings (at the 4-leaf stage). Emitters were positioned 3 cm from the plant base. In T1 and T3 plots, two randomly selected emitters were equipped with tensiometers installed at 20 cm depth to monitor soil matric potential. Irrigation was initiated across all plots when any monitored value dropped below -35 kPa, with a single irrigation quota of 10 mm. The total irrigation water amount (W) during the entire tomato growing season was 4200 m³·ha^-1^, 3900m³·ha^-1^, and 4000 m³·ha^-1^ in 2021, 2022, and 2023, respectively. The fertilization regime involved weekly fertigation using a compound fertilizer (N-P_2_O_5_-K_2_O = 20-20-20 + trace elements). If irrigation thresholds were not met, one-third of the irrigation quota (3.3 mm) was applied solely for fertilization, corresponding to a fertilizer application rate of 61.11 kg·ha^-^¹ per session.

Field management practices included topping tomato plants when the fifth fruit cluster developed. The tomato growth cycle was divided into stages (seedling, flowering, fruit setting, rapid expansion, and maturation), with standardized protocols for weed control, pest management (using low-residue pesticides), pruning, and vine training. All procedures were rigorously repeated across growing seasons to ensure experimental consistency.

### 2.3 Measured parameters and methods

(1) Greenhouse temperature monitoring: Air temperature and humidity were monitored using a hygrothermograph (BENETECH GM1365, China) suspended 1.2 m above ground in the experimental area. Data were automatically recorded at 1-hour intervals.

(2) Plant growth parameters: At maturity each year, three randomly selected plants per treatment were analyzed. Plant height was measured from the soil surface to the apex of the main stem using a tape measure. Stem diameter was determined at 10 cm above the soil surface using a vernier caliper (Zou et al., 2017). For dry matter accumulation measurement, three plants per treatment were deactivated in a 105°C oven for 30 min, then dried to constant mass at 75°C. Dry mass was quantified using a 0.001 g precision analytical balance (Du et al., 2020).

(3) Tomato yield and its components: During each growing season, the fruit number per truss (*N_f_*) on individual plants was recorded periodically. At maturity, fruits were harvested in multiple batches to determine the average single fruit weight *W_f_* (g), measured to the nearest 0.1 g. The yield per plant *Y_p_* (kg) was calculated using Equation 1:


(1) $$Y_{P}\;=\frac{W_{f}\times N_{f}}{1000}$$

The final total tomato yield T*_y_* (10^4^ kg·ha^-1^) is converted through Equation 2. Wherein, the number of plants per plot is *N_p_* = 20, and *A* is the plot area (m²).


(2) $$T_{y}=\frac{\;Y_{p}\;\times N_{p}\times 10^{4}}{A}\;$$

(4) Crop water productivity (CWP) calculation was calculated using Equation 3 as follows:


(3) $$CWP=T_{y}/I\;$$

Where CWP is the Crop water productivity, kg·m^-^³; T_y_ is the total output of tomatoes, kg·ha^-1^;I represents the total irrigation amount, m³·ha^-1^.

(5) Tomato quality assessment: At fruit maturity, ten uniformly sized fruits at equivalent ripening stages were selected from the 2nd and 3rd trusses of plants within each treatment. A composite homogenate was prepared for analysis by pooling one-quarter of the pericarp tissue from each sampled fruit. All determinations were performed with three analytical replicates per parameter. Lycopene content: Quantified by ultraviolet-visible (UV-Vis) spectrophotometry (Hu et al., 2005). Soluble solids content (SSC): Measured using a digital refractometer (precision: ± 0.1°Brix).Soluble sugar content: Determined via the anthrone-sulfuric acid colorimetric assay (Zhang and Li, 2016).Titratable acidity (TA): Assessed by acid-base titration (Zhang and Li, 2016). Sugar-acid ratio: Calculated as soluble sugar content divided by titratable acidity. Ascorbic acid (vitamin C) content: Measured by 2,6-dichlorophenolindophenol (DCPIP) titration (Zhang and Li, 2016).

Among the aforementioned tomato fruit quality parameters, the contents of soluble sugars, sugar-acid ratio, soluble solids content (SSC), vitamin C (ascorbic acid), and lycopene exhibit a positive correlation with quality (i.e., higher values indicate superior quality). To quantitatively evaluate fruit quality, a relative scoring system was established: All quality parameters for the CK (control) treatment were assigned a baseline relative value of 100. For each subsequent treatment, the relative value of a specific quality parameter was calculated as the ratio of its measured content in that treatment to the corresponding value in the CK treatment (Equation 4):


(4) $$q_{r}=\frac{q_{i}}{q_{ck}}$$

In the formula, q_i_ represents the relative score of the i-th quality indicator (soluble sugars, sugar-acid ratio, soluble solids content (SSC), ascorbic acid (vitamin C), or lycopene). wi is the weighting coefficient (all assigned 0.2). Organic acids were not included in the evaluation system due to the lack of clear superiority. Then the overall quality Q of tomato fruit can be expressed as shown in Equation 5:


(5) $$Q=\sum_{i=1}^{5}\left(wi\times \frac{q_{i}}{q_{ck}}\right)$$

(6) Soil chemical indicators: At the end of each year’s test, soil samples from the 0–20 cm layer were randomly collected, air-dried, and extracted at a soil-water ratio of 1:5 (mass ratio) with 3 minutes of stirring. Electrical conductivity (EC) was measured using a benchtop conductivity meter (Leici DDS-307A, China), and pH was determined with a pH meter (Leici PHS-2F, China). Soil organic matter (SOM) content was calculated via the potassium dichromate oxidization-ferrous sulfate titration method (Ministry of Agriculture of the People’s Republic of China [MOA], 2006). Soil available potassium was analyzed by the neutral ammonium acetate extraction-flame photometric method (MOA, 2004), hydrolysable nitrogen by the alkaline diffusion method (Mulvaney and Khan, 2001), and available phosphorus by sodium bicarbonate extraction-molybdenum-antimony anti-spectrophotometry (MOA, 2014).

### 2.4 Data analysis

Data were organized in Microsoft Excel (Microsoft Corp., USA) and visualized using Origin 2024 Pro (OriginLab Corp., USA). Statistical analyses, including one-way ANOVA and two-way ANOVA (to assess biochar rate [C], year [Y], and interaction [C×Y]), were performed in SPSS 27.0 (IBM Corp., USA). *Post hoc* comparisons used Fisher’s LSD test (P< 0.05).

## 3 Results and analysis

### 3.1 Effects of biochar dosage on soil physicochemical properties

**Figure 2:**
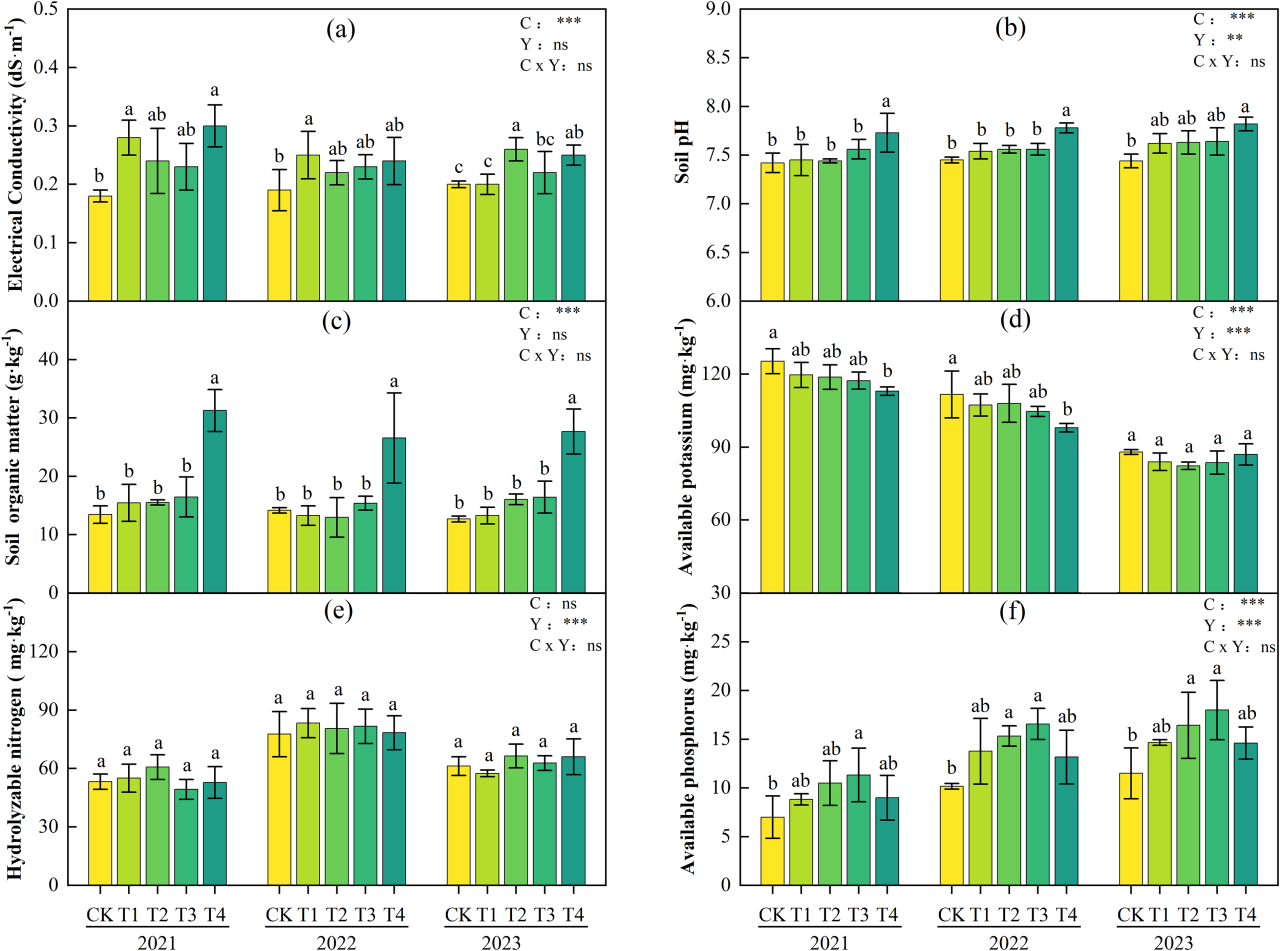
Soil chemical properties (electrical conductivity **(a)**; pH **(b)**; organic matter **(c)**; available potassium **(d)**; hydrolyzable nitrogen **(e)**; available phosphorus **(f)**) in the 0–20 cm soil layer under different biochar treatments during 2021-2023. Different lowercase letters indicate significant differences among treatments at P< 0.05 (applies similarly to subsequent tables/figures). According to LSD testing, F-values and significance levels for biochar (C), year (Y), and their interaction (C × Y) were calculated at P = 0.05. Significance is denoted as: *P< 0.05, **P< 0.01, ***P< 0.001; ns, not significant.

**Figure 2** presents the soil chemical properties in the 0–20 cm soil layer after tomato harvest during 2021-2023. While soil EC values increased with biochar application compared to the CK treatment, they remained at low levels (non-saline). Significant increases in soil pH and organic matter content were observed only in the T4 treatment, with maximum increases of 0.4 units and 17.83 g·kg^-1^ (132.8%), respectively. The differences in soil available potassium content among treatments showed a decreasing trend annually, becoming non-significant by the third year. Although differences in soil hydrolyzable nitrogen content existed among treatments, they were not significant. Soil available phosphorus content significantly increased in 2 and T3 treatments, with maximum increases of 50.8% and 63.0%, respectively. Two-way ANOVA (**Figure 2**) showed that biochar application rate (C) had significant main effects on soil pH, EC, available potassium, available phosphorus, and SOM content. Interannual variation (Y) significantly influenced soil pH, hydrolyzable nitrogen, available phosphorus, and available potassium. Interaction effect analysis revealed that biochar and interannual variation had highly significant interactions (P< 0.01) only for soil pH, EC, and available potassium, with no significant interaction effects on SOC, hydrolyzable nitrogen, or available phosphorus.

### 3.2 Effects of biochar application rate on tomato growth

**Figure 3:**
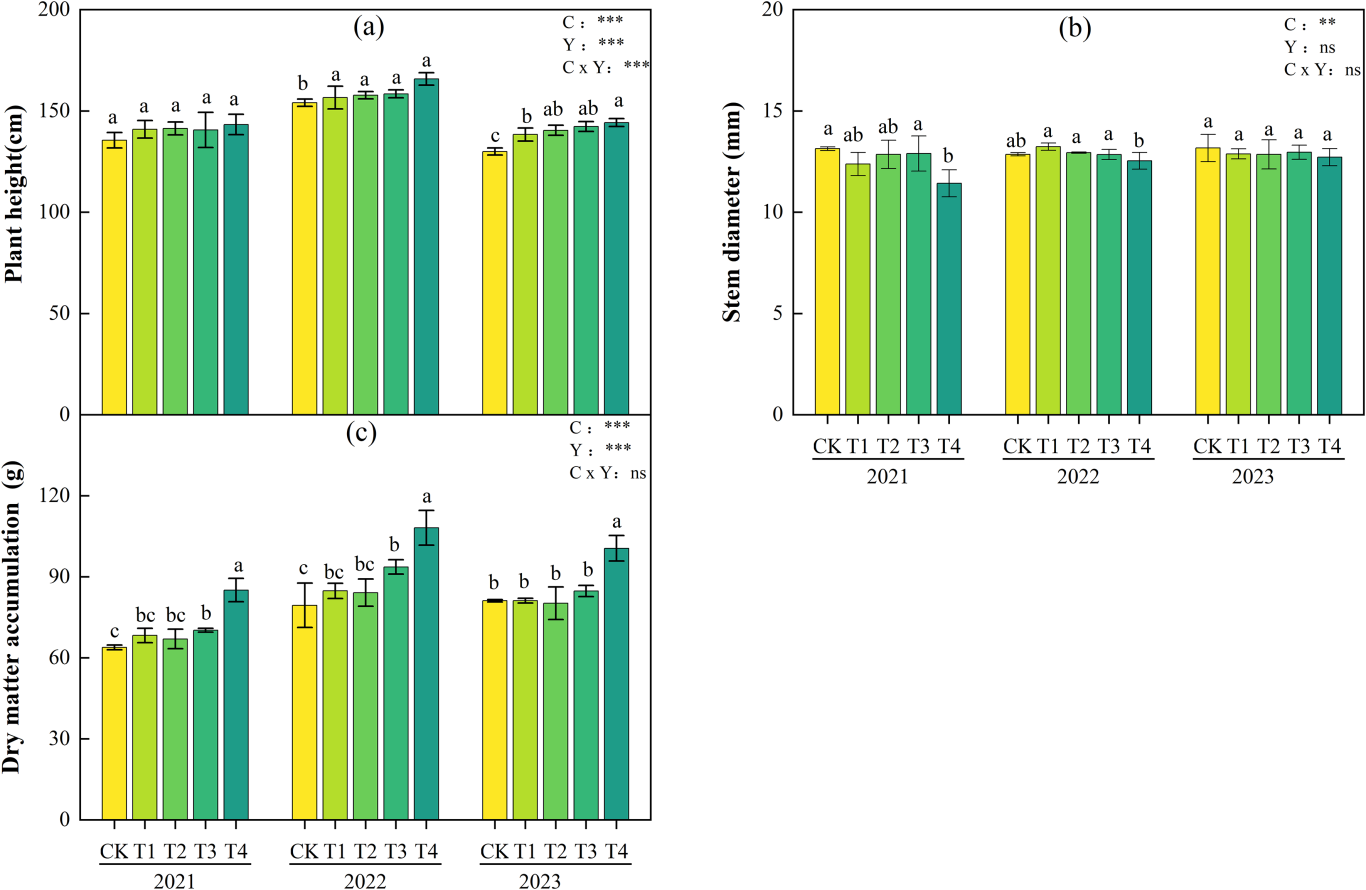
Plant height **(a)**, stem diameter **(b)**, and plant dry mass **(c)** of tomatoes under different biochar application rates during 2021-2023. Different lowercase letters indicate significant differences among treatments at P< 0.05. According to LSD testing, F-values and significance levels for biochar (C), year (Y), and their interaction (C × Y) were calculated at P = 0.05. Significance is denoted as: *P< 0.05, **P< 0.01, ***P< 0.001; ns, not significant.

**Figure 3** displays the growth status of tomatoes under different biochar application rates. As the biochar application rate increased, both plant height and dry matter accumulation of tomatoes showed a gradual increasing trend. Compared with CK, the plant height in the T4 treatment increased by 5.7%, 7.1%, and 11.0% in 2021, 2022, and 2023, respectively, and the aboveground dry matter accumulation increased by 33.2%, 36.1%, and 23.9%, with significant differences (P< 0.05). Except that the stem diameter of the T4 treatment was significantly smaller than that of the CK treatment in 2021, there were no significant differences in stem diameter between the T1-T4 treatments and the CK treatment in other years.

Two-way ANOVA results (**Figure 3**) showed that biochar application rate (C) had significant or highly significant effects on plant height, stem diameter, and dry matter accumulation. Interannual variation (Y) had highly significant effects on plant height and dry matter accumulation. The interaction between biochar and interannual variation had no significant effects only on stem diameter and dry matter accumulation, indicating that the differences in tomato growth indices across years were primarily driven by the independent effects of biochar application rate and year, rather than their interaction.

### 3.3 Effects of biochar application rate on tomato yield and its components

**Figure 4:**
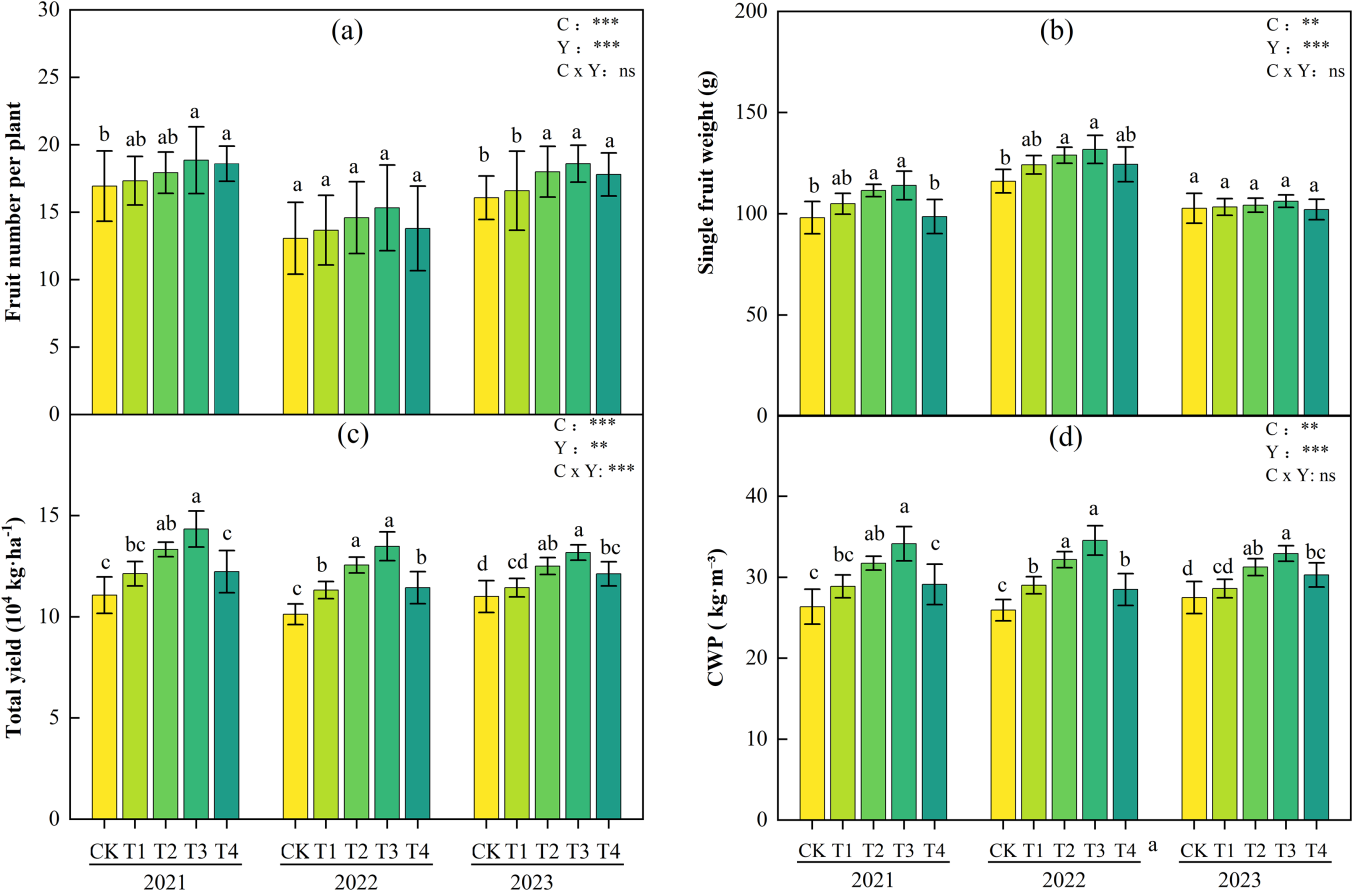
Fruit number per plant **(a)**, single fruit weight **(b)**, total yield **(c)**, and Crop water productivity(CWP) **(d)** of tomatoes under different biochar application rates during 2021-2023. Different lowercase letters indicate significant differences among treatments at P< 0.05. According to LSD testing, F-values and significance levels for biochar (C), year (Y), and their interaction (C × Y) were calculated at P = 0.05. Significance is denoted as: *P< 0.05, **P< 0.01, ***P< 0.001; ns, not significant.

**Figure 4** presents the tomato yield and its components under different biochar application rates during 2021-2023. The yield parameters (fruit number per plant, single fruit weight, total yield) exhibited significant dose-response relationships to biochar application rates (C) across growing seasons (P< 0.05). Over three years, T1-T4 treatments significantly increased fruit number per plant, single fruit weight, and total yield compared with CK, but no interannual cumulative effects were observed. The highest yield and its components were recorded in T3. Compared with CK, T3 significantly increased fruit number per plant by 11.4%, 17.4%, and 15.8%, single fruit weight by 16.3%, 13.5%, and 3.5%, and yield by 29.5%, 33.2%, and 19.8% in 2021, 2022, and 2023, respectively (P< 0.05). T2 ranked second, with significant increases in fruit number per plant (5.9%, 11.7%, 12.0%), single fruit weight (13.7%, 11.1%, 1.5%), and yield (20.4%, 24.1%, 13.7%) compared with CK across the three years (P< 0.05). Meanwhile, CWP in T1-T4 treatments showed a synchronous increasing trend with yield indicators.

Two-way ANOVA results (**Figure 4**) showed that the main effects of biochar application rate (C) and interannual variation (Y) on fruit number per plant, single fruit weight, total yield, and CWP were all highly significant (P< 0.01). However, the C×Y interaction effects on single fruit weight, fruit number per plant, and CWP were not significant. Notably, the C×Y interaction had a significant effect on tomato yield, indicating that the yield-enhancing effect of biochar is influenced by interannual environmental factors.

### 3.4 The influence of biochar dosage on tomato quality

**Figure 5:**
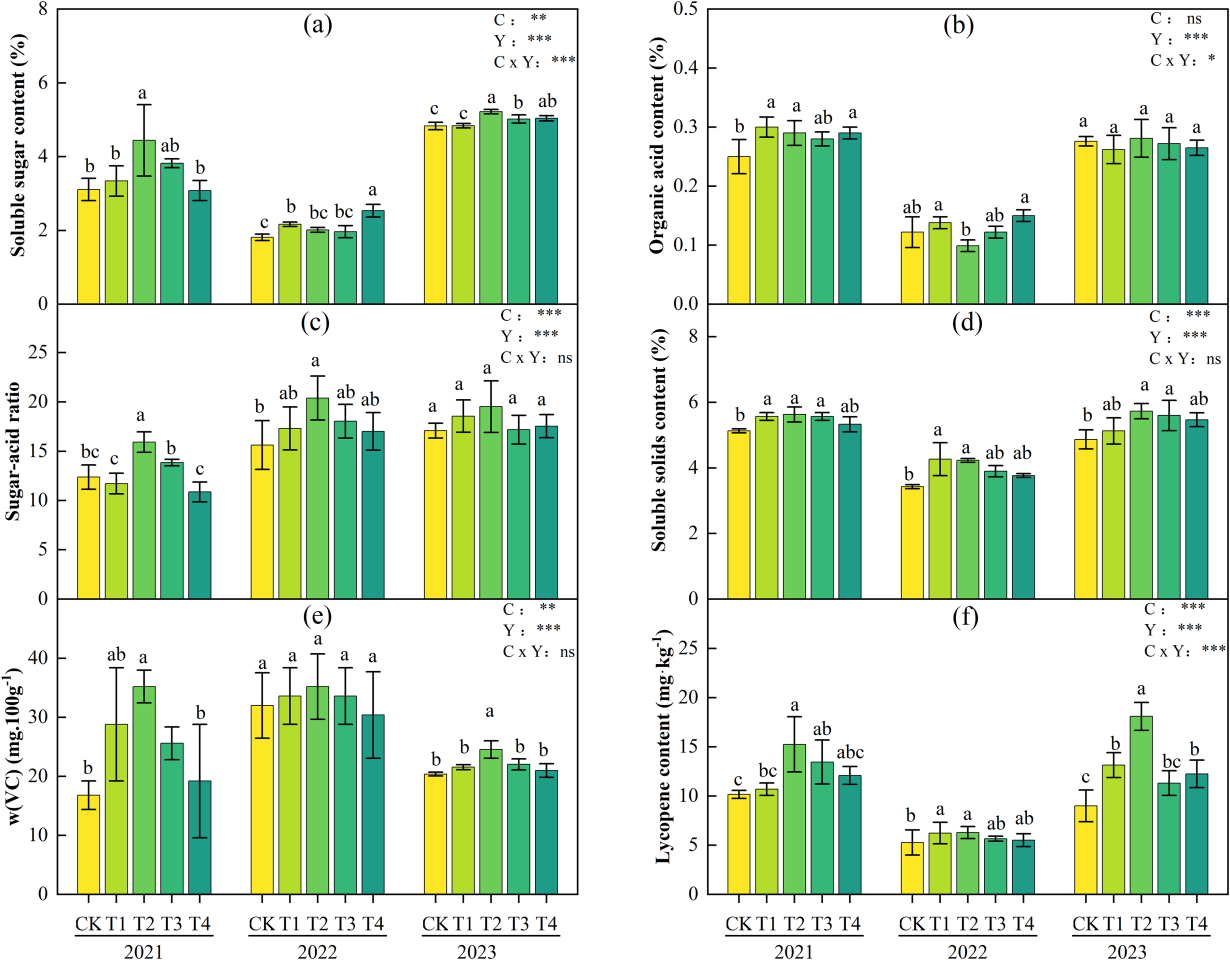
Soluble sugar content **(a)**, organic acid content, **(b)** sugar-acid ratio **(c)**, total soluble solids (TSS) content **(d)**, VC **(e)** and lycopene content **(f)** of tomatoes under different biochar treatments during 2021-2023. Different lowercase letters indicate significant differences among treatments at P< 0.05. According to LSD testing, F-values and significance levels for biochar (C), year (Y), and their interaction (C × Y) were calculated at P = 0.05. Significance is denoted as: *P< 0.05, **P< 0.01, ***P< 0.001; ns, not significant.

**Figure 5** shows the quality indicators of tomato fruits treated with different biochar application rates from 2021 to 2023. It can be seen that the dose-effect relationship was basically the same over the three years, that is, with the increase of biochar application rate, the contents of soluble sugar, sugar-acid ratio, vitamin C (VC), and lycopene in tomatoes all showed a single-peak dose-response characteristic of rising first and then falling. The maximum values mostly occurred in the T2 treatment. Compared with the CK treatment, the soluble sugar content increased significantly by 43.1%, 11.0%, and 8.0% respectively in 2021-2023, and the sugar-acid ratio increased by 28.7%, 30.5%, and 14.2% respectively, reaching significant levels in the first two years. VC content increased by 109.5%, 10.0% and 20.7% respectively; Lycopene content increased significantly by 50.07%, 19.35%, and 101.36%. Followed by T1 treatment, The maximum increases in soluble sugar, sugar-to-acid ratio, vitamin C (VC), and lycopene in 2021–2023 were 19.4%, 10.8%, 71.4%, and 46.2%, respectively. However, excessive application of biochar (4.0 kg·m^-^², T4 treatment) can cause fluctuations or even reductions in quality indicators, such as soluble sugar and sugar-acid ratio in 2021 and VC content lower than CK in 2022, suggesting a tendency for excessive biochar application to have inhibitory effects on individual fruit quality.

Two-way ANOVA results (**Figure 5**) showed that biochar application rate (C) and interannual variation (Y) had significant main effects on soluble sugar, sugar-acid ratio, TSS, lycopene, and VC content. In terms of interaction effects, C×Y significantly influenced soluble sugar and lycopene, while the interaction effects on sugar-acid ratio, TSS, and VC were not significant, with their responses primarily driven by the independent effects of biochar application rate or year.

**Figure 6:**
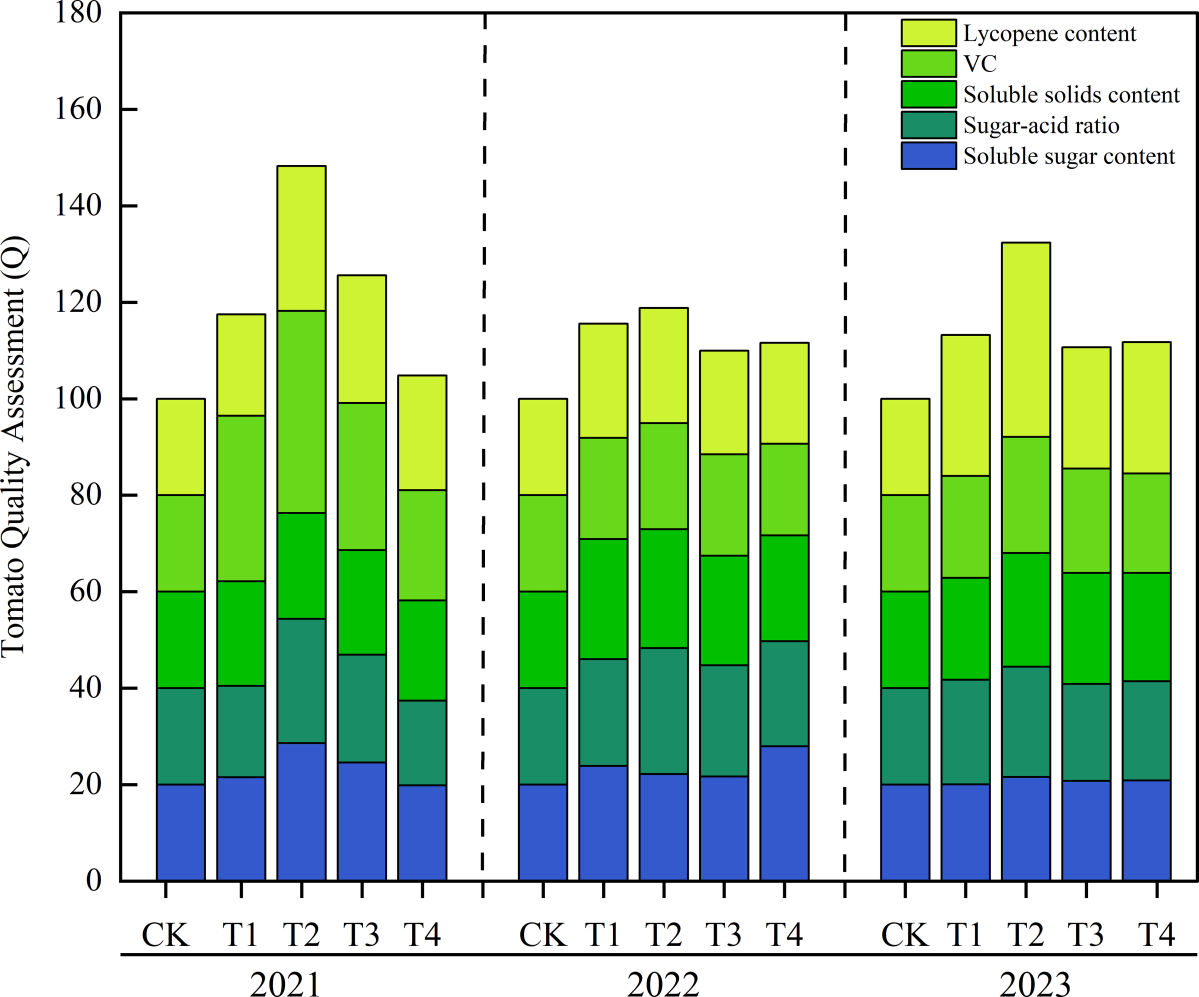
Comprehensive evaluation of tomato quality attributes with different biochar amendments over three consecutive years.

As shown in **Figure 6**, the comprehensive quality index score (Q) of tomato fruits reached the maximum value in the T2 treatment from 2021-2023, increasing by 48.23%, 18.82%, and 32.36% compared with the CK treatment, respectively. The three-year average values of comprehensive quality indices across treatments showed that compared with CK, the comprehensive scores of T1-T4 increased by 15.4%, 33.1%, 15.4%, and 9.4%, respectively, with the order: T2 > T1 = T3 > T4 > CK.

### 3.5 Correlations between tomato yield/quality parameters and soil chemical properties

**Figure 7:**
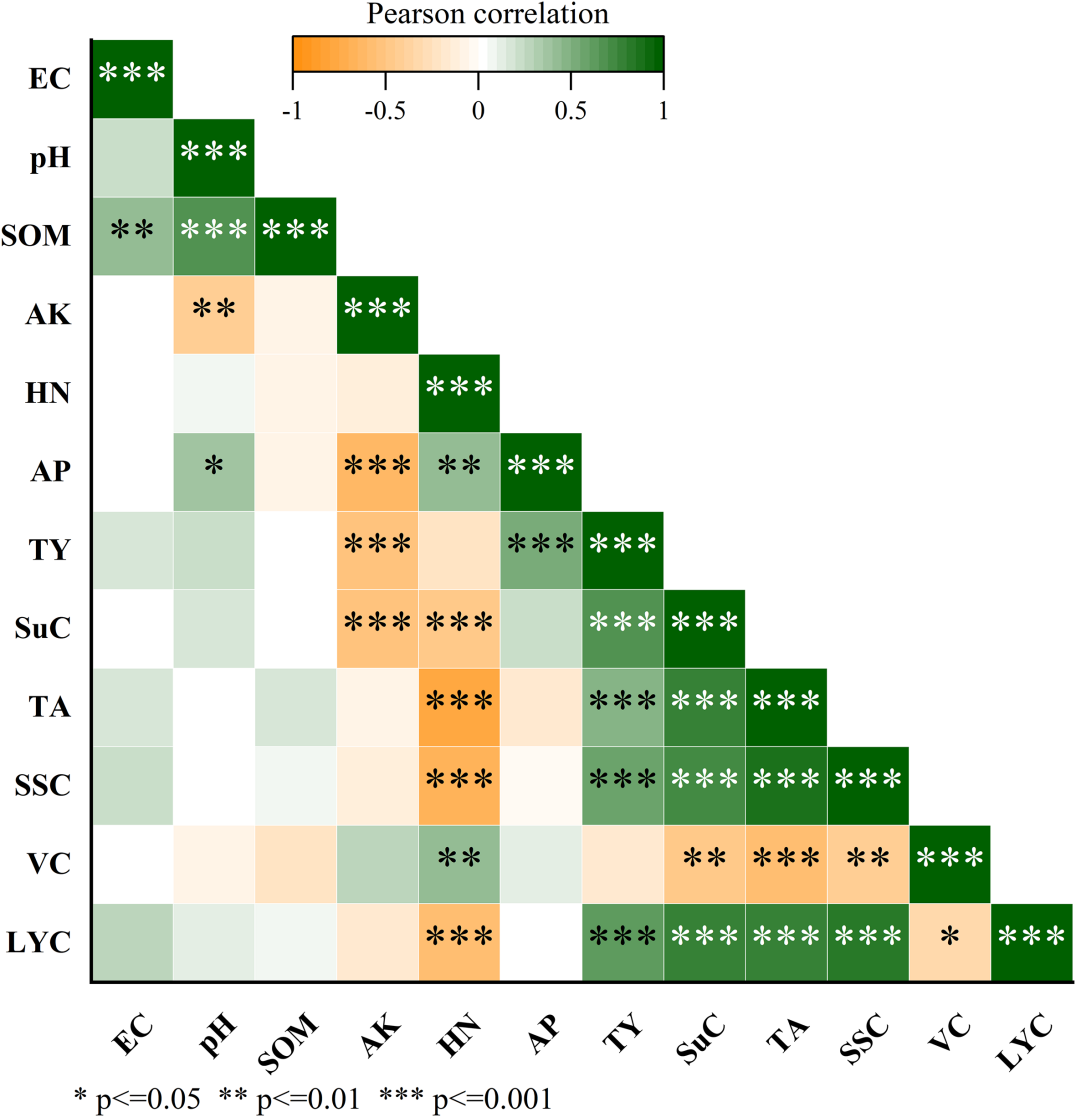
Pearson correlation analysis of tomato yield, quality parameters and soil chemical properties. EC=Electrical Conductivity (dS·m^-1^), pH=Soil pH, SOM=Soil organic matter (g·kg^-1^), AK=Available potassium (mg·kg^-1^), HN=Hydrolyzable nitrogen ( mg·kg^-1^), AP=Available phosphorus (mg·kg^-1^), TY=Tomato yield (10^4^ kg·ha^-1^), SuC=Soluble sugar content (%), TA=Organic acid content (%), SSC=Soluble solids content (%), VC=w(VC) (mg.100g^-1^), LYC=Lycopene content (mg·kg^-1^).

The Pearson correlation matrix constructed based on the data from a 3-year biochar application rate experiment (**Figure 7**) systematically reveals the correlation between soil chemical indices and tomato yield and quality at the end of the experiment. Firstly, the tomato yield (TY) shows an extremely significant positive correlation with soil available phosphorus (AP) (r = 0.41, p ≤ 0.01), indicating that when the content of AP in the soil is high, the absorption and utilization of phosphorus by plants are not inhibited; on the contrary, sufficient supply of available phosphorus may promote yield increase, confirming that phosphorus is the core factor driving yield. In contrast, TY has an extremely significant negative correlation with available potassium (AK) (r = -0.63, p ≤ 0.01). A high residual content of AK in the soil means that plants absorb less potassium, which may inhibit yield formation due to an imbalance between potassium supply and demand. Secondly, the soluble sugar content (SuC) has extremely significant negative correlations with both soil available potassium (AK) and hydrolyzable nitrogen (HN) (AK: r = -0.55, p ≤ 0.01; HN: r = -0.49, p ≤ 0.01), indicating that the higher the contents of AK and HN in the soil, the less potassium and nitrogen are absorbed by plants, which restricts carbohydrate synthesis and leads to a decrease in SuC. Organic acid (TA), soluble solids content (SSC), and lycopene (LYC) all show extremely significant negative correlations with soil HN (TA: r = -0.76, p ≤ 0.001; SSC: r = -0.69, p ≤ 0.001; LYC: r = -0.57, *p ≤ 0.001), suggesting that a high content of HN in the soil results in less nitrogen absorption by plants, thus inhibiting the synthesis of the above substances. However, vitamin C (VC) has a significant positive correlation with HN (r = 0.41, p ≤ 0.01), implying that when the residual nitrogen content in the soil is high, although plants absorb and utilize less nitrogen, it may promote VC synthesis through specific metabolic pathways, reflecting the differential regulatory effect of nitrogen on quality indices. In addition, soil EC, pH, and total organic matter (TOM) show no significant correlations with tomato yield and quality.

## 4 Discussion

### 4.1 Effects of biochar on soil physicochemical properties

The regulatory effects of biochar on soil nutrient cycling are closely related to its own physicochemical properties. In this study, biochar application increased soil pH, EC, and SOM content, which is consistent with most acidic soil improvement studies (Zhang et al., 2025). This is because biochar itself has a high pH (usually alkaline) and contains alkaline cations (e.g., Ca²^+^, Mg²^+^), which neutralize H^+^ in the soil through release processes, thereby driving pH elevation (Schulz et al., 2013). The increase in soil EC may be caused by a dual mechanism: 1) Direct salt input from dissolution of biochar-derived ash (Mao et al., 2024). 2) The increase in soil pH increases the dissociation degree of weakly acidic functional groups such as carboxyl and phenolic hydroxyl groups, generating more negative charges (Wang et al., 2014), thereby enhancing the adsorption and exchange capacity of soil colloids for cations, promoting the release of cations from mineral salts, increasing ion concentration in the soil solution, and leading to an increase in EC (Łuczak et al., 2021). The continuous accumulation of SOM is primarily attributed to three aspects: 1) The decomposition rate of organic carbon input by biochar is significantly lower than its application amount, promoting long-term SOC accumulation (Jin et al., 2019); 2) The abundant pore structure of biochar provides physical protection space for organic molecules, enhancing their adsorption and retention capacity for active organic matter (Yang et al., 2025); 3) Biochar increases microbial carbon use efficiency, causing microorganisms to use more organic carbon for biosynthesis rather than respiration (Kalu et al., 2024a), thereby indirectly inhibiting soil microbial respiration and reducing the organic carbon mineralization rate (Weng et al., 2017).In this study, biochar application was observed to decrease soil available potassium content, increase soil available phosphorus content, and cause no significant change in hydrolyzable nitrogen. The reverse response of available potassium may be related to the cation competition effect of high-temperature pyrolyzed biochar. The increased organic matter content and cation exchange capacity (CEC) by biochar enhanced the adsorption competition between potassium ions and cations such as calcium and magnesium, leading to a decrease in the available potassium content (He et al., 2025). The increase in soil available phosphorus content is because biochar application can stimulate phosphorus-solubilizing bacteria (Gul and Whalen, 2016). Additionally, different amounts of biochar can inhibit acid phosphatase activity while enhancing alkaline phosphatase activity, promoting P hydrolysis and ensuring a continuous supply of available phosphorus in the soil (Peng et al., 2023; Zhao et al., 2020). Although some studies have shown that biochar can increase soil total nitrogen storage (Ding et al., 2016), after biochar is applied to the soil, its high C/N ratio causes microorganisms to absorb more nitrogen from the soil for their own growth and metabolism during the decomposition of organic matter in biochar, thereby reducing the hydrolyzable nitrogen content in the soil (Nguyen et al., 2017). However, some studies have shown that the amount of extractable nutrients in the soil can increase after biochar application (Wang et al., 2024). This discrepancy may be related to fertilizer types, soil types, and microbial activities (Burrell et al., 2016). Additionally, structural characteristics of biochar such as pore size distribution and surface functional groups can also affect its regulatory effects on soil nutrient cycling (Parasar and Agarwala, 2025). In practical applications, these influencing factors may act synergistically to jointly determine the regulatory effects of biochar on soil nutrient cycling.

### 4.2 Effects of biochar on tomato growth and yield

The yield-enhancing effects of biochar on crops have been widely documented (Agbna et al., 2017). Our study further demonstrates that appropriate biochar application rates significantly promoted tomato plant height, dry matter accumulation, and yield formation. The underlying mechanisms include: 1) biochar increases soil CEC by releasing H^+^ through abundant oxygen-containing functional groups and adsorbing cations in the soil (Kabir et al., 2023). The porous network structure of biochar further enhances cation adsorption, thereby improving soil nutrient supply capacity, promoting root proliferation and nutrient absorption, and increasing plant height and dry matter accumulation; 2) biochar improves soil physical structure and water-holding capacity, strengthens water use by reducing evaporation and increasing transpiration (Xiao et al., 2024), and enhances CWP, thus increasing fruit yield. 3) biochar application improved the soil microenvironment by promoting soil enzyme activity and microbial abundance, making more nutrients available for tomato plants (Guo et al., 2021). However, under continuous annual biochar application, no dose-dependent cumulative yield effects were observed. This may be because the soil system reached a dynamic equilibrium after long-term biochar addition, where further increases in application rate did not sustainably enhance crop growth once soil physical, chemical, and biological properties were optimized. In 2021, the T4 treatment (4.0 kg·m^-^²) exhibited significantly reduced stem diameter and diminished yield gains. This could be attributed to excessive biochar causing a significant decline in soil available potassium, combined with potential plant growth inhibitors (low-content incompletely pyrolyzed volatile organic compounds) (Deenik et al., 2010), which may have inhibited soil nitrogen-fixing microbial activity or directly impacted root development, thereby exerting negative effects on crop growth at high application rates (Obadi et al., 2023).

Compared with previous studies, the effects of biochar at different rates on tomato yield in this study exhibited certain discrepancies. For example, Guo et al. (2021) found that 35 t·ha^-1^ biochar (3.5 kg·m^-^²) maximized tomato yield under greenhouse conditions, while Lei et al. (2024) reported a linear increase in yield with increasing biochar application rates. Such differences may originate from variations in biochar feedstock, pyrolysis temperature, soil baseline properties, and experimental environments. For instance, She et al. (2018) demonstrated that biochars pyrolyzed at different temperatures significantly differed in their effects on tomato growth and yield.

### 4.3 Effects of biochar on tomato fruit quality

This study demonstrates that the application of biochar at suitable rates significantly enhances multiple quality indices of tomato fruits. Specifically, the soluble sugar content of tomatoes in biochar-treated plots increased significantly. This improvement can be attributed to the following mechanisms: biochar application promotes root growth, enhances plant nutrient uptake capacity, facilitates the translocation of photosynthates, and thereby accelerates carbohydrate accumulation in fruits—ultimately leading to higher soluble sugar content (Yan et al., 2024; Mazzurco-Miritana et al., 2025). The sugar-acid ratio, an important parameter for evaluating tomato taste, significantly increased in biochar-treated plots compared with CK, indicating that biochar synergistically regulated organic acid metabolism while increasing soluble sugars, balancing fruit flavor. Total soluble solids (TSS) content also increased with biochar application rates, consistent with reports by Almaroai and Eissa (2020). The mechanism may involve biochar-improved soil structure promoting water homeostasis, thereby enhancing fruit dry matter accumulation efficiency (Ikram et al., 2024).Vitamin C (VC), a key non-enzymatic antioxidant, represents a major nutritional quality indicator of tomatoes (Giannakourou and Taoukis, 2021). Experimental results showed VC content increased to varying degrees with biochar application, consistent with findings by Guo et al. (2021) and Agbna et al. (2017). This is attributed to biochar enhancing rhizospheric soil enzyme activity and improving root environment. Root growth plays a crucial role in nutrient uptake, significantly influencing vitamin C content in plants (Yang et al., 2020). Additionally, lycopene content in biochar-treated tomatoes was significantly higher than CK in all years. Previous studies by Khan et al. (2019) under field conditions reported increased antioxidant compounds (ascorbic acid, lycopene, β-carotene) in ripe tomato fruits with biochar application, consistent with our results. However, Petruccelli et al. (2015) reported conflicting results, possibly due to differences in test regions, soil types, and biochar types (Suthar et al., 2018).

Two-way ANOVA showed highly significant interannual main effects (P< 0.01) on all tomato quality indices, likely due to interannual environmental fluctuations altering plant photosynthetic efficiency and secondary metabolism, thereby modifying quality expression (Devadze et al., 2025). Specifically, microclimatic differences across the three growing seasons drove varying quality responses: the 2022 season had a mean temperature of 26.0 °C and relative humidity of 70.1%, and its specific environmental conditions notably inhibited photosynthesis and secondary metabolism during fruit development—explaining the relatively lower accumulation of sugars, organic acids, and lycopene, and thus weaker quality improvements that year (Zheng et al., 2023). While 2023 had the highest mean temperature (27.1 °C), its lower relative humidity (64.2%) and more favorable diurnal temperature variation likely alleviated the impacts of adverse environmental conditions. In contrast, 2021 maintained relatively moderate conditions (mean temperature 25.7 °C, relative humidity 67.4%), which were more conducive to quality formation.

### 4.4 Cumulative effects of continuous biochar application

After three years of continuous biochar application in this experiment, soil nutrient cycling gradually stabilized, with no significant dose accumulation effect observed. The reasons can be attributed to the following aspects: Firstly, as the application period increases, the saturation effect of soil adsorption sites limits the continuous retention capacity of biochar (Kalu et al., 2024a). Meanwhile, biochar forms stable complexes with soil minerals and organic matter, causing some nutrients to be trapped inside these complexes and reducing their bioavailability (Kalu et al., 2024a), which in turn weakens the sustained promotional effect on soil fertility and crop growth. Secondly, the aging process of biochar alters its surface chemical properties, reducing its nutrient adsorption capacity and reactive activity, thereby weakening its regulatory effect on soil nutrient cycling (Apostolović et al., 2024). Thirdly, long-term biochar application can induce adaptive changes in soil microorganisms (Idbella et al., 2024). Microorganisms show improved efficiency in utilizing carbon sources and nutrients in biochar, and may influence the transformation and supply of soil nutrients through feedback regulatory mechanisms. Simultaneously, microbial metabolites or enzymes can either inhibit or promote the transformation of different nutrients, further complicating the regulatory effect of biochar (Sharma et al., 2025). Ultimately, these factors result in an insignificant dose accumulation effect.

## 5 Conclusions

Collectively, the three-year fixed-location field trial demonstrated that compared with the CK treatment, biochar-amended treatments (T1-T4) increased tomato fruit number per plant and average fruit weight to varying degrees, thereby enhancing overall yield formation. Notably, the T2 and T3 treatments achieved the most substantial and statistically significant (P< 0.05) yield increments, ranging from 13.7% to 24.1% and 19.8% to 33.2%, respectively. Fruit quality analysis revealed that the T2 treatment yielded tomatoes with the highest comprehensive quality index, followed by T1 and T3. The three-year mean comprehensive quality scores for these treatments increased by 33.1%, 15.4%, and 15.4%, respectively. No significant interannual cumulative effects of biochar application rate on crop yield or quality were observed. In conclusion, a biochar application rate of 1.0-2.0 kg·m^-^² optimally achieves synergistic enhancement of both yield and fruit quality in greenhouse tomato production systems.

Future research could focus on the following directions: 1) Deciphering key functional microbial groups in biochar-microbe-plant interactions through high-throughput sequencing; 2) Revealing metabolic pathways of biochar regulating fruit quality formation by integrating transcriptomics; 3) Conducting multi-region and multi-crop long-term field experiments to evaluate the long-term effects of biochars with different feedstocks and pyrolysis temperatures on soil quality, nutrient cycling, and crop productivity. These studies will provide more systematic theoretical support for the precise application of biochar in sustainable protected agriculture.

## Data Availability

The raw data supporting the conclusions of this article will be made available by the authors, without undue reservation.
